# Triple X syndrome: Psychiatric disorders and impaired social functioning as a risk factor

**DOI:** 10.1192/j.eurpsy.2022.2355

**Published:** 2022-12-21

**Authors:** Maarten Otter, Bea C. M. Campforts, Constance T. R. M. Stumpel, Thérèse A. M. J. van Amelsvoort, Marjan Drukker

**Affiliations:** 1 Department of Psychiatry and Neuropsychology, School for Mental Health and Neuroscience, Maastricht University, Maastricht, The Netherlands; 2 Department of Community Mental Health in Mild Intellectual Disabilities, Trajectum, Zutphen, The Netherlands; 3 Medical Department, SIZA, Arnhem, The Netherlands; 4 Department of Clinical Genetics and School for Oncology and Developmental Biology, Maastricht University Medical Centre, Maastricht, The Netherlands

**Keywords:** Impaired social functioning, prevalence of psychiatric disorders, triple X syndrome, X chromosome

## Abstract

**Background:**

Women with triple X syndrome (TXS) have an extra X chromosome. TXS appeared to be associated with psychiatric disorders in biased or underpowered studies.

**Aim:**

This study aims to describe the prevalence of psychiatric disorders in adults with TXS in a relatively large and less biased group of participants.

**Method:**

In this cross-sectional study, data were collected from 34 women with TXS (mean age = 32.9; s.d. = 13.1) and 31 controls (mean age = 34.9; s.d. = 13.7). Psychiatric disorders were assessed using the MINI International Neuropsychiatric Interview (MINI) and the adult behavior checklist (ABCL). Trait and state anxiety were assessed using the State–Trait Anxiety Inventory.

**Results:**

In the TXS group, MINI results showed a higher prevalence of major depressive episodes (43.3%), psychotic disorders (29.4%), and suicidality (23.5%). Only 50% of the TXS group earned a normal score for the total syndrome score using the ABCL. In addition, levels of trait anxiety were higher in the TXS group. Only three women in each group received psychotropic medication. Impaired social functioning appeared to represent a major risk factor in TXS as regards psychotic, affective disorders, trait anxiety, and low self-esteem.

**Conclusions:**

Women with TXS are vulnerable to developing psychiatric disorders, and women with both TXS and impaired social functioning are even more vulnerable.

## Background

Knowledge with respect to the increased prevalence of psychiatric disorders in adult women diagnosed with triple X syndrome (TXS) is scarce [1]. Psychiatric disorders and psychological complaints, as well as the modifier impaired social functioning, are hardly studied in this group.

Modern psychiatrists are increasingly aware of the contribution of genetics to the etiology of psychiatric disorders. Genetics is a compulsory subject during the training to become a psychiatrist [2]. Psychiatric disorders, like psychotic disorders and impaired social functioning, may be associated to copy number variants [3]. So today, psychiatrists may likely refer for genetic evaluation. Genetic evaluation of people with a psychotic disorder and impaired social functioning may reveal copy number variants but also a 47,XXY karyotype in men (Klinefelter syndrome) [4] or a 47,XXX karyotype in women [5,6]. These women with an extra X chromosome have so-called TXS, a genetic condition that occurs in 1 out of 1,000 women. TXS was first described in 1959 [7]. Women with TXS who already know about their genetic condition—for example, after a prenatal diagnosis—may seek psychiatric expertise, for example, in specialist units for patients with genetic and neurodevelopmental disorders. However, there is almost no scientific literature on psychiatric problems in adult women with TXS. In 1973, Staffan Olanders, a Swedish psychiatrist, wrote his doctoral thesis on 39 women with an extra X chromosome and described different types of hallucinations, delusions, and affective disturbances, including suicidality, impaired social functioning, and behavioral disorders. The fact that he recruited the majority of his participants in mental hospitals created a highly biased group of participants [8]. Nevertheless, there is evidence that women with psychotic disorders have a four times higher prevalence of TXS than the general population [9]. In 2004, genetic evaluation was performed in 12 patients with mild learning disabilities and psychiatric disorders in our department, and TXS was diagnosed in three adult female patients and one adolescent girl. We subsequently performed a literature search on TXS [10] and found no systematic studies in unbiased groups of women with TXS and psychiatric disorders. In a case report, we described the psychopathology of two of these women [6]. They showed slightly decreased intelligence levels, psychotic disorders, impaired social functioning, suicidal ideations, traumatic experiences, affective disorders, and low self-esteem. More recently, Freilinger et al. [1] described symptoms of psychological distress in girls and women with TXS, and reported that half of them showed no behavioral or social deficits. Women without (*n* = 20) and with (*n* = 12) impaired social functioning were described in a previous report from our group [5]. A Danish nationwide study of hospital diagnoses and prescribed psychiatric medication in an unselected cohort of women with TXS (*n* = 103) demonstrated that women with TXS have an increased risk to develop psychiatric conditions [11]. In the current study on psychiatric disorders and psychological complaints, we analyzed the differences between these two groups.

## Aim

The present study aims to address the research gap in the description of psychiatric disorders in adult women with TXS. In this study, we examined the same group of 34 women with TXS and 31 controls, as described previously [5,12,13]. First, we compared psychopathology between the TXS-group and the control group. Second, we assessed the contribution of Full-Scale IQ (FSIQ) to the levels of psychopathology. Third, we compared the risk of psychopathology in TXS women with impaired social functioning to those without it, because people with impaired social functioning are more at risk to develop psychiatric disorders, for example, psychotic disorders [14,15].

## Methods

### Participants

Sixty-five adult (≥18 years) women participated in the study, 34 with TXS (47,XXX karyotype) and 31 controls. In order to be eligible to participate in this study, subjects had to be capable of giving informed consent and had to be sufficiently proficient in the Dutch language.

### Recruitment

Participants with TXS were recruited through flyers, digital newsletters, social media, the Dutch TXS support group, advertising, and the Department of Clinical Genetics of Maastricht University Medical Centre (MUMC+). The control group was recruited through families and friends of women with TXS and advertising. To lower the barrier to entry into the study, we encouraged the women with TXS to be accompanied by a friend or relative who participated in the study in several cases. This study was part of a larger research project on neuroimaging, neuropsychology, and neuropsychiatry in adults with TXS. When possible, all assessments were performed on the same day to make it as easy as possible for the participants. The data were collected between 2015 and 2018. Two women with TXS under legal guardianship, did not meet the inclusion criteria.

### Study design and setting

This study was a cross-sectional study comparing a group of adults with TXS with a control group.

### Measures

FSIQ was assessed using a shortened version of the Dutch Wechsler Adult Intelligence Scale, third edition (WAIS-III) [16]. Psychopathology was assessed using the clinician rated MINI International Neuropsychiatric Interview (Dutch version; DSM-IV) (MINI) [17,18] and the Dutch authorized and tested version of the adult behavior checklist (ABCL) [19], the adult version of the child behavior checklist. The MINI was used to interview the participants, and the ABCL was completed by peer informants, like family members or friends. The MINI [17] has been developed as a short structured interview for the most relevant psychiatric disorders (axis I) in the diagnostic and statistical manual of mental disorders—fourth edition (DSM-IV) [20]. The structured MINI interview allows administration by nonspecialized interviewers. The assessments were done by psychologists and medical students after their theoretical and practical training in psychiatry and training on the job. The students worked under the supervision of experienced research assistants. According to the DSM-IV, criteria for post-traumatic stress disorder requires past traumatic experiences and current re-experiencing. As traumatic experiences were described in TXS [6], results of traumatic experiences and current re-experiencing will be presented. The MINI interview does not provide details concerning traumatic experiences. However, some participants disclosed details about these experiences spontaneously.

The ABCL is an instrument to assess psychopathology in the general population and has been developed to be completed by proxy respondents. The ABCL is suitable for the 18- to 59-year age group. The ABCL includes 132 behavioral problems items which were evaluated for the preceding 6 months. Behavioral problem statements were scored by a peer informant on a three-level rating scale (“not true,” “somewhat or sometimes true,” and “very true”). Six DSM-oriented scales and eight syndrome scales were identified. The “internalizing syndrome scale” was derived from a summary score from the withdrawn, somatic complaints, and anxious/depressed syndrome scales. Similarly, the “externalizing syndrome scale” was derived from the rule-breaking behavior and aggressive behavior syndrome scales. The “total problem score” was derived from the sum of all syndrome scales. Item 79 on speech problems and item 91 on suicide talk were assessed separately.

Self-esteem was assessed using the ABCL items 33 (feels unloved), 35 (feels worthless), 47 (lacks self-confidence), and 107 (can’t succeed). We combined these items to assess self-esteem.

Trait and state anxiety were assessed using the State–Trait Anxiety Inventory-Dutch version (STAI) [21,22]. The STAI has 20 items for assessing trait anxiety and 20 items for state anxiety. All items were rated on a four-point Likert scale; higher scores indicated increased anxiety levels. The STAI has been developed in nonclinical samples and thus provides scores on anxiety levels that do not necessarily reach the strict cut-off levels from the DSM [23], but give essential information on mental health.

Social functioning was assessed using the Social Responsiveness Scale-Adults version (SRS-A) as described in a previous report [5]. This report described the results of the SRS-A in the TXS group in four classes: high functioning (*n* = 1), normal functioning (*n* = 19), mild-to-moderate deficits (*n* = 7), and severe deficits (*n* = 5). Because of the small numbers in some of the categories, we dichotomized social functioning into two groups, one with and one without impairments in social functioning.

Data on the use of medical compounds were collected.

### Statistical analyses

To assess differences in psychopathology between the TXS and the control group, normally distributed continuous variables data were compared between the TXS group and the control group using the Student’s *t*-test (T-scores for the ABCL internalizing, externalizing, and total syndrome scales). Differences in categorical variables (MINI, raw scores of the ABCL) were analyzed using Fisher’s exact test.

In order to assess the contribution of FSIQ to the levels of psychopathology, the association between TXS and the ABCL T-scores were analyzed using linear regression analysis adjusting for FSIQ. In order to assess impaired social functioning as a risk factor for the development of psychiatric disorders, MINI scores, ABCL raw scores, and T-scores of the internalizing, externalizing, and total syndrome scores were analyzed in the TXS women with impaired social functioning in comparison to those without impaired social functioning.

All statistical analyses were performed using STATA/MP for Mac, version 13.1 (StataCorp, College Station, TX). All analyses were two-tailed, and alpha was set at 0.05. This study has an exploratory nature. Therefore, correction for multiple testing was not performed [24]. Bonferroni correction would set alpha at 0.0007.

## Results

### Participant characteristics

The age of the participants (18–63 years of age) was relatively similar between the TXS group (*n* = 34) and the control group (*n* = 31), with mean age of 32.9 and 34.9 years (standard deviation [s.d.] = 13.1 and 13.7), respectively (*t*(63) = −0.59, *p* = 0.56). Mean FSIQ was significantly lower in TXS subjects than in controls (mean [*M*] = 86.1, s.d. = 10.5, 95% CI 82.3–89.9 vs *M* = 96.8, s.d. = 12.7, 95% CI 92.1–101.4, *p* = 0.0005). Among the 34 women with TXS, 10 were diagnosed prenatally (mean age = 26.1 years, s.d. = 9.1), while the remaining 24 were diagnosed postnatally. The indications for postnatal testing included infertility/recurrent abortions (*n* = 9; mean age = 44.3, s.d. = 9.4), atypical development (*n* = 6; mean age = 28.5, s.d. = 11.5), history of a family member with a genetic condition (*n* = 4; mean age = 45.8, s.d. = 11.7), small head (*n* = 2), intestinal malformation (*n* = 1), nuchal edema (*n* = 1), and epicanthal folds (*n* = 1). A total of 73.5 and 80% of the participants in the TXS and control groups were premenopausal at the time of the data collection. The number of the participants that used psychotropic medication was three in the TXS and three in the control group.

### Psychiatric disorders in the TXS group compared to the control group

The frequency of lifetime psychotic disorders and major depressive episodes (MDEs) was higher in the TXS group than in the control group (Cramér’s V = −0.41; *p* = 0.001 and Cramér’s V = −0.34; *p* = 0.011, respectively; Table 1). Concerning suicidality, 17.65% of the TXS group reported past attempts and 23.5% of the TXS group reported a current suicidal risk. There was no difference between the groups in relation to substance abuse related disorders. In the TXS group and the control group, 60.6 and 29.3%, respectively, reported traumatic experiences (Cramér’s V = −0.32; *p* = 0.014). However, current re-experiencing showed only minor differences (in the TXS group 35% vs 22.2% in the control group, data not shown). Some participants disclosed that the traumatic experiences concerned sexual abuse (*n* = 3) or bullying (*n* = 3).

**Table 1.** MINI neuropsychiatric interview in comparison to the TXS group and the control group.

	TXS *n* = ‥/‥ (‥%)	Controls *n* = ‥/‥ (‥%)	*p-*value^a^	Cramér’s V
Psychotic disorders current	5/34 (14.7%)	0/31 (0.0%)	0.054	−0.28
Psychotic disorders lifetime	10/34 (29.4%)	0/31 (0.0%)	**0.001**	−0.41
Major depressive episode: current	4/34 (11.8%)	0/31 (0.0%)	0.12	−0.24
Major depressive episode: past	13/30 (43.3%)	4/31 (12.9%)	**0.011**	−0.34
Dysthymia	4/33 (12.1%)	0/31 (0.0%)	0.11	−0.25
Suicidality: past attempts	6/34 (17.65%)	0/31 (0.0%)	**0.026**	−0.30
Suicidal risk current	8/34 (23.5%)	1/31 (3.2%)	**0.028**	−0.29
Hypomania	5/34 (14.7%)	1/31 (3.2%)	0.20	−0.20
Mania	2/34 (5.8%)	0/31 (0.0%)	0.49	−0.17
Generalized anxiety disorder	7/34 (20.6%)	1/31 (3.2%)	0.056	−0.26
Panic disorder: lifetime	5/34 (14.7%)	0/31 (0.0%)	0.054	−0.28
Obsessive–compulsive disorder	2/34 (5.9%)	1/31 (3.2%)	1.0	−0.06
Post-traumatic stress disorder	0/34 (0.0%)	0/31 (0.0%)		

a Fisher’s exact test.

*Abbreviations:* MINI, MINI International Neuropsychiatric Interview; TXS, triple X syndrome.

The results of the ABCL (Table 2) showed statistically significant differences in relation to internalizing problems and total problems, but not externalizing problems. Thought problems in the ABCL syndrome scale were indicative of psychotic disorders (Cramér’s V = −0.39; *p* = 0.004). The DSM oriented scores on depressive problems showed statistically significant differences, but anxious problems did not (Table 2). The assessment of self-esteem in the ABCL revealed differences between the two groups, with higher scores on low self-esteem in the TXS group (Cramér’s V = −0.59; *p* = 0.001). Speech problems (ABCL item 79) were significantly more often reported in the TXS group (Cramér’s V = −0.41; *p* = 0.002; data not shown), but “suicide talks” (ABCL item 91) were not (Cramér’s V = −0.24; *p* = 0.147; data not shown). This might indicate that women with TXS avoid talking about their suicidal feelings and thoughts.

**Table 2.** Summary of group differences of ABCL results in the triple X syndrome (TXS) and control groups.

	TXS group (*n* = 33)			Control group (*n* = 31)			*p*-value^a^	Cramér’s V
	Normal range *n* = ‥ (‥%)	Borderline range *n* = ‥ (‥%)	Clinical range *n* = ‥ (‥%)	Normal range *n* = ‥ (‥%)	Borderline range *n* = ‥ (‥%)	Clinical range *n* = ‥ (‥%)		
ABCL DSM-oriented scales								
Depressive problems	16 (48.48%)	8 (24.24%)	9 (27.27%)	26 (83.87%)	5 (16.13%)	0	**0.001**	−0.43
Anxiety problems	27 (81.82%)	2 (6.06%)	4 (12.12%)	28 (90.32%)	3 (9.68%)	0	0.161	−0.25
Avoidant pers. problems	16 (48.48%)	5 (15.15%)	12 (36.36%)	30 (96.77%)	1 (3.23%)	0	**<0.001**	−0.54
Somatic problems	22 (66.67%)	4 (12.12%)	7 (21.21%)	27 (87.10%)	1 (3.23%)	3 (9.68%)	0.158	−0.25
Inattention	27 (81.82%)	6 (18.18%)	0	28 (90.32%)	2 (6.45%)	1 (3.23%)	0.259	−0.22
Hyperactivity–impulsivity	24 (72.73%)	4 (12.12%)	5 (15.15%)	27 (87.10%)	4 (12.90%)	0	0.103	−0.28
ADHD	25 (75.76%)	3 (9.09%)	5 (15.15%)	27 (87.10%)	3 (9.68%)	1 (3.23%)	0.355	−0.20
Antisocial problems	27 (81.82%)	4 (12.12%)	2 (6.06%)	29 (93.55%)	2 (6.45%)	0	0.432	−0.20
ABCL syndrome scale								
Thought problems	22 (66.67%)	8 (24.24%)	3 (9.09%)	30 (96.77%)	1 (3.23%)	0	**0.004**	−0.39
Anxious depressed	24 (72.73%)	2 (6.06%)	7 (21.21%)	28 (90.32%)	2 (6.45%)	1 (3.23%)	0.087	−0.27
Withdrawn	20 (60.61%)	5 (15.15%)	8 (24.24%)	28 (90.32%)	3 (9.68%)	0	**0.007**	−0.39
Somatic complaints	21 (63.64%)	3 (9.09%)	9 (27.27%)	27 (87.10%)	0	4 (12.90%)	0.051	−0.30
Attention problems	22 (66.67%)	7 (21.21%)	4 (12.12%)	29 (93.55%)	2 (6.45%)	0	**0.020**	−0.35
Aggressive behavior	28 (84.85%)	3 (9.09%)	2 (6.06%)	28 (90.32%)	3 (9.68%)	0	0.592	−0.17
Rule-breaking behavior	27 (81.82%)	2 (6.06%)	4 (12.12%)	31 (100%)	0	0	0.039	−0.31
Intrusive	27 (81.82%)	3 (9.09%)	3 (9.09%)	29 (93.55%)	2 (6.45%)	0	0.236	−0.22
Internalizing	13 (39.39%)	4 (12.12%)	16 (48.48%)	23 (74.19%)	4 (12.90%)	4 (12.90%)	**0.007**	−0.39
Externalizing	21 (63.64%)	5 (15.15%)	7 (21.21%)	25 (80.65%)	3 (9.68%)	3 (9.68%)	0.328	−0.19
Total syndrome score	16 (48.48%)	5 (15.15%)	12 (36.36%)	23 (74.19%)	6 (19.35%)	2 (6.45%)	**0.015**	−0.36

a Fisher’s exact test.

*Abbreviations:* ABCL, adult behavior checklist; ADHD, attention deficit hyperactivity disorder; DSM, diagnostic and statistical manual of mental disorders.

The TXS group reported higher levels of anxiety as assessed with the STAI in comparison to the control group. This was at the time of the interview (TXS STAI: *M* = 34.3; s.d. = 10.0; 95% CI = 30.7, 37.8 vs control group: *M* = 30.2; s.d. = 4.6; 95% CI = 28.5, 31.9; *t*(62) = 2.07; *p* = 0.042) as well as in the weeks before the interview (TXS STAI: *M* = 42.6; s.d. = 11.9; 95% CI = 38.4, 46.8; vs control group: *M* = 33.3; s.d. = 8.9; 95% CI = 30.1, 36.6; *t*(62) = 3.51; *p* = 0.0008).

When we controlled for FSIQ as a potential confounder, partial eta-squared (η_p_^2^) values of the internalizing problems (η_p_^2^ = 0.18), externalizing (η_p_^2^ = 0.04), and total problems (η_p_^2^ = 0.13) appeared to be low. This means that the differences between the TXS and the control groups were only partially explained by differences in FSIQ.

### Psychiatric disorders in women with TXS without and with impaired social functioning

MINI results (Table 3) in relation to psychotic disorders showed the strongest association (Cramér’s V = 0.45), so women with TXS as well as impaired social functioning more often suffer from psychotic disorders than women with TXS without impaired social functioning. ABCL DSM oriented results (Table 4) showed the strongest association in relation to anxiety problems (Cramér’s V = 0.62), inattention (Cramér’s V = 0.62), and antisocial problems (Cramér’s V = 0.62), so women with TXS and impaired social functioning more often suffer from anxiety, inattention, and antisocial problems. The ABCL syndrome scales (Table 4) showed strong associations between the prevalence of anxiety, attention deficit hyperactivity disorder (ADHD) related behavior, somatic complaints, and behavioral problems and impaired social functioning. The TXS group without impaired social functioning showed higher levels of self-esteem (*M* = 2.9; s.d. = 1.5; 95% CI = 2.2, 3.6; data not shown) in comparison with the TXS group with impaired social functioning (*M* = 4.7; s.d. = 2.1; 95% CI = 3.4, 6.1; *t*(30) = −2.86; *p* = 0.0076; data not shown). Speech problems showed small differences (Cramér’s V = 0.36; *p* = 0.12; data not shown). In contrast, the results in relation to “suicide talks” (item 91) showed major differences (Cramér’s V = 0.56; *p* = 0.004; data not shown).

**Table 3.** MINI neuropsychiatric interview: comparison between the TXS without and with social impairments.

	TXS with no social impairments *n* = ‥/‥ (‥%)	TXS with (mild) social impairments *n* = ‥/‥ (‥%)	*p-*value^a^	Cramér’s V
Psychotic disorders current	1/20 (5.0%)	4/12 (33.3%)	0.053	0.38
Psychotic disorders lifetime	3/20 (15.0%)	7/12 (58.3%)	**0.018**	0.45
Major depressive episode: current	1/20 (5.0%)	3/12 (25.0%)	0.14	0.29
Major depressive episode: past	7/19 (36.8%)	5/9 (55.6%)	0.43	0.18
Dysthymia	1/20 (5.0%)	3/11 (27.3%)	0.12	0.32
Suicidality: past attempts	3/20 (15.0%)	3/12 (25.0%)	0.65	0.12
Suicidal risk current	4/20 (20.0%)	3/12 (25.0%)	1.00	0.06
Hypomania	2/20 (10.0%)	3/12 (25.0%)	0.34	0.20
Mania	0/12 (0.0%)	2/12 (16.7%)	0.13	0.33
Generalized anxiety disorder	2/20 (10.0%)	5/12 (41.7%)	0.073	0.37
Panic disorder: lifetime	1/20 (5.0%)	4/12 (33.3%)	0.054^a^	0.38
Obsessive–compulsive disorder	2/20 (10.0%)	0/12 (3.2%)	0.52^a^	0.20
Post-traumatic stress disorder	0/20 (0.0%)	0/11 (0.0%)		

a Fisher’s exact test.

*Abbreviations:* MINI, MINI International Neuropsychiatric Interview; TXS, triple X syndrome.

**Table 4.** Summary of group differences of ABCL results in the TXS without and with social impairments.

	TXS without social impairments (*n* = 20)			TXS with (mild) social impairments group (*n* = 12)			*p*-value^a^	Cramér’s V
	Normal range *n* = ‥ (‥%)	Borderline range *n* = ‥ (‥%)	Clinical range *n* = ‥ (‥%)	Normal Range *n* = ‥ (‥%)	Borderline Range *n* = ‥ (‥%)	Clinical range *n* = ‥ (‥%)		
ABCL DSM-oriented scales								
Depressive problems	13 (65.00%)	4 (20.00%)	3 (15.00%)	2 (16.67%)	4 (33.33%)	6 (50.00%)	0.023	0.49
Anxiety problems	20 (100.00%)	0	0	6 (50.00%)	2 (16.67%)	4 (33.33%)	**0.001**	0.62
Avoidant pers. problems	12 (60.00%)	3 (15.00%)	5 (25.00%)	3 (25.00%)	2 (16.67%)	7 (58.33%)	0.130	0.36
Somatic problems	16 (80.00%)	2 (10.00%)	2 (10.00%)	5 (41.67%)	2 (16.67%)	5 (41.67%)	0.077	0.41
Inattention	20 (100%)	0	0	6 (50.00%)	6 (50.00%)	0	**0.001**	0.62
Hyperactivity–impulsivity	17 (85.00%)	3 (15.00%)	0	6 (50.00%)	1 (8.33%)	5 (41.67%)	**0.006**	0.56
ADHD	18 (90.00%)	2 (10.00%)	0	6 (50.00%)	1 (8.33%)	5 (41.67%)	**0.005**	0.56
Antisocial problems	20 (100.00%)	0	0	6 (50.00%)	4 (33.33%)	2 (16.67%)	**0.001**	0.62
ABCL syndrome scale								
Thought problems	16 (80.00%)	2 (10.00%)	2 (10.00%)	5 (41.67%)	6 (50.00%)	1 (8.33%)	**0.045**	0.45
Anxious depressed	19 (95.00%)	1 (5.00%)	0	4 (33.33%)	1 (8.33%)	7 (58.33%)	**<0.001**	0.70
Withdrawn	14 (70.00%)	3 (15.00%)	3 (15.00%)	5 (41.67%)	2 (16.67%)	5 (41.67%)	0.236	0.31
Somatic complaints	15 (75.00%)	1 (5.00%)	4 (20.00%)	5 (41.67%)	2 (16.67%)	5 (41.67%)	0.167	0.34
Attention problems	16 (80.00%)	4 (20.00%)	0	5 (41.67%)	3 (25.00%)	4 (33.33%)	**0.014**	0.51
Aggressive behavior	19 (95.00%)	1 (5.00%)	0	8 (66.67%)	2 (16.67%)	2 (16.67%)	0.074	0.40
Rule-breaking behavior	19 (95.00%)	1 (5.00%)	0	7 (58.33%)	1 (8.33%)	4 (33.33%)	**0.007**	0.50
Intrusive	19 (95.00%)	1 (5.00%)	0	7 (58.33%)	2 (16.67%)	3 (25.00%)	**0.018**	0.48
Internalizing	11 (55.00%)	2 (10.00%)	7 (35.00%)	1 (8.33%)	2 (16.67%)	9 (75.00%)	**0.021**	0.47
Externalizing	16 (80.00%)	4 (20.00%)	0	4 (33.33%)	1 (8.33%)	7 (58.33%)	**<0.001**	0.68
Total syndrome score	13 (65.00%)	3 (15.00%)	4 (20.00%)	2 (16.67%)	2 (16.67%)	8 (66.67%)	**0.014**	0.50

a Fisher’s exact test.

*Abbreviations:* ABCL, adult behavior checklist; ADHD, attention deficit hyperactivity disorder; DSM, diagnostic and statistical manual of mental disorders; MINI, MINI International Neuropsychiatric Interview; TXS, triple X syndrome.

The TXS group without impaired social functioning reported lower levels of anxiety as assessed with the STAI (*M* = 31.6; s.d. = 9.3; CI = 27.3, 36.0) in comparison to the TXS group with social impairments (*M* = 39.4; s.d. = 10.3; CI = 32.5, 46.4; *t*(29) = −2.15; *p* = 0.04) at the time of the interview and also lower levels of anxiety in the weeks before the interview (the TXS group without social impairments: *M* = 39.0; s.d. = 12.4; CI = 33.3, 44.8; vs the TXS group with social impairments: *M* = 49.5; s.d. = 9.1; CI = 43.4, 55.7; *t*(29) = −2.46; *p* = 0.02). In summary, women with TXS and impaired social functioning more often have psychiatric disorders and psychological complaints than women with TXS without social impairments.

## Discussion

### Main findings and comparison with findings from other studies

Only 50% of the TXS group earned a normal score in the ABCL total syndrome score, and even a smaller part of the TXS group scored normal in the internalizing syndrome score (39.4%; Table 2). The results of the MINI interview revealed a much higher prevalence of past MDEs (43.3%), lifetime psychotic disorders (29.4%), and current suicidality (23.5%) (Table 1). As opposed to a more extended version of the MINI (the MINI Plus), the MINI cannot differentiate between the subtypes of psychotic disorders, like delusional or schizophrenic disorders. The results of the current psychotic disorders and current melancholic features (Table 1) may not be representative, as it was not expected that subjects with current melancholic features would join this study as a participant. The STAI revealed higher levels of anxious complaints in the TXS group (*t*(62) = 3.51; *p* = 0.0008).

In cases with lifetime psychotic disorders, impaired social functioning (Cramér’s V = 0.45; *p* = 0.018) appeared to be a risk factor to the women with TXS (Table 3). The ABCL internalizing (Cramér’s V = 0.47; *p* = 0.021), like the anxious/depressed syndrome scores (Cramér’s V = 0.70; *p* ≤ 0.001) and externalizing syndrome scores including antisocial and rule-breaking behavior (Cramér’s V = 0.68; *p* ≤ 0.001), revealed higher levels of psychopathology in the group of women with TXS and impaired social functioning.

Previous studies recruited smaller groups of participants [1] or suffered from higher levels of recruitment bias as in Olanders [8]. Freilinger (2018) showed that 50% of a small group of adult women with TXS function without psychiatric disorders, which is in accordance with our results. Our previous report on two cases with TXS demonstrated slightly decreased intelligence levels, psychotic disorders, impaired social functioning, suicidal ideations, traumatic experiences, affective disorders, and low self-esteem [6]. The current study adds to the knowledge of TXS syndrome that impaired social functioning appear to represent a risk factor in TXS as regards psychotic, affective disorders, attentional problems, and low self-esteem, but not in relation to traumatic experiences and suicidality. Attentional problems has been discussed in more detail in another report from our group on neuropsychological findings in the same group of participants [13]. Decreased levels of FSIQ appear not to represent a significant risk factor. Importantly, we observed severe medical undertreatment, as only three women in the TXS group (*n* = 34) and three in the control group (*n* = 31) received psychotropic medication Table 5. This is in contrast with the findings of the Danish nationwide study that described increased prescriptions in the TXS group of psycholeptic drugs (26.2% in the TXS group and 20.7% in the control group), antipsychotics (11.7% in the TXS group and 5.5% in the control group), psychoanaleptic drugs (29.1% in the TXS group and 20.7% in the control group), antidepressant drugs (26.2% in the TXS group and 17.3% in the control group), and ADHD medication and nootropics (3.9% in the TXS group and 1.5% in the control group) [11]. This can be explained by the fact that the Danish study collected data from medical settings. In the Netherlands, the care for people with genetic disorders like TXS is mainly provided by psychologists as the first tier of healthcare providers. Psychologists—of course—prefer nonmedical treatments and are not allowed to prescribe medication in the Netherlands. Furthermore, it is our experience that women with TXS are reluctant to use psychiatric medication.

**Table 5.** Current medications.

Medication class	Number in TXS (*n* = 34) (%)	Number in controls (*n* = 31) (%)
Psychopharmacologic medications	3 (8.8%)	3 (9.7%)
ADHD medication	1 (2.9%)	0
Antidepressants	2 (5.9%)	1 (3.2%)
Antipsychotics	2 (5.9%)	0
Mood stabilizers	1 (2.9%)	1 (3.2%)
Tranquilizers	2 (5.9%)	2 (6.5%)
Other	2 (5.9%)	0
Medication for physical disorders (except contraceptives or vitamins)	20 (58.8%)	15 (48.4%)
Anticonvulsants	1 (2.9%)	0
Analgesic drugs	7 (20.6%)	3 (9.7%)
Medication to treat migraine	3 (8.8%)	1 (3.2%)
Respiratory medications	8 (23.5%)	9 (29.0%)
Antihistamines	7 (20.6%)	7 (22.6%)
Gastrointestinal medications for constipation	5 (14.7%)	0
Antacids	6 (17.6%)	4 (12.9%)
Contraceptive hormonal treatment	12 (35.3%)	14 (45.2%)
Thyroid hormones	0	3 (9.7%)
Diabetes medication	0	1 (3.2%)
Cholesterol medications	1 (2.9%)	1 (3.2%)
Dermatological ointments	3 (8.8%)	1 (3.2%)
Anticoagulant drugs	2 (5.9%)	1 (3.2%)
Antirheumatic drugs	3 (8.8%)	0
Hypertension medication	0	2 (6.5%)
Vitamins	9 (26.5%)	4 (12.9%)

*Abbreviations:* ADHD, attention deficit hyperactivity disorder; TXS, triple X syndrome.

### Implications

Clinicians who work with women with TXS should be aware of the risk of psychiatric disorders, including psychotic disorders, affective disorders and suicidality. Clinicians should be aware of the possibility of a not yet recognized TXS diagnosis, especially those who work with women with psychotic disorders and impaired social functioning. It is essential to consider that women with TXS may suffer from expressive language disorders. A structured clinical interview like MINI can help uncover all complaints, including somewhat embarrassing ones such as traumatic experiences or suicidal thoughts, that were not already shared with the clinician. Improving the psychological and psychiatric diagnostic procedures will therefore have to lead to improvement of psychotherapeutic treatments and treatment with psychiatric medication in accordance with the guidelines.

### Strengths, limitations, and how to design future studies

Our group focuses on research on adult women with TXS. Several groups have been investigating sex chromosomal disorders without enough attention to the features of the distinct disorders [25–27]. This way, the special needs of women with TXS are underestimated. Moreover, previous studies mainly focus on children with sex chromosomal disorders [25–27]. The study of TXS is far behind the study of other sex chromosomal disorders. Even in 2019, a review of sex chromosomal disorders and psychiatric disorders mentions that women with TXS are not at risk of developing impaired social functioning [28]. These differences may be explained by the lack of physical features in TXS. Clinicians seldom suspect a TXS diagnosis based on external or endocrinological characteristics, contrasting with Turner and Klinefelter syndrome [29]. Therefore, this study is important in filling the gap in our knowledge of psychiatric disorders in TXS.

This descriptive and explorative study has limitations. Our group of adults with TXS was more extensive and less biased than any other sample, but the numbers were relatively small. As TXS is not rare, it should be possible to establish larger groups of participants and investigate them in a longitudinal design. The number of participants in the current study was too small to search for differences in various age groups and for differences between premenopausal and postmenopausal women with TXS. A longitudinal design also offers the opportunity to identify early markers of “at risk” development and assess early interventions’ effectiveness [26]. As soon as early interventions appear to be effective, early recognition of TXS is the next step, probably by noninvasive prenatal testing [30] or screening of every newborn and subsequent counseling of the parents [31] and potentially preventing psychological problems. Another limitation of this study is the assessment of self-esteem with an unvalidated tool.

Despite the small numbers, this study may suggest new themes to focus on in future studies. The present paper dichotomized social functioning, but this is not a dichotomous construct. Studies in larger groups are necessary to investigate differences in the prevalence of psychopathology between groups with various levels of social functioning. The study of self-esteem, until now seldom recognized as a biologically based personality feature [32], deserves further study. Furthermore, the previously described variability in self-esteem in people with psychotic disorders deserves further scientific attention in the study of TXS, preferably in an ecological study design [15,33]. Comparably, suicidality in TXS deserves further scientific attention, which could be helpful to women with TXS, but also could extend the knowledge about genetic and other factors that contribute to suicidal thoughts and behavior [34].

Klinefelter syndrome has been cited as a genetic model of psychotic disorders in men [35]. TXS may provide a unique model to study psychotic disorders associated with impaired social functioning, which is important as both may interact and may be associated with poor daily life outcomes in women [36]. The relation between the extra X chromosome and psychiatric disorders in TXS remains to be elucidated. Several pathogenetic mechanisms have been hypothesized. The extra X chromosome might cause decreased cell-division rates [10], which might explain the smaller head circumference and the decreased total brain volumes in TXS [37–39]. Gene dosage imbalances in X chromosomal genes that escape X chromosome inactivation [40–43] and autosomal genes [42–45] might also play a role in the pathophysiological process from the extra X chromosome to neurobiological disturbances and the subsequent psychiatric disorders. Several X-linked genes have been mentioned as candidate genes [46]. These studies addressed fundamental scientific issues concerning the biology of sex chromosomes. They yielded several new candidate genes to be explored in future studies because some are linked to mental retardation and brain development [42,43]. However, these interesting studies did not find an explanation for the variability of the psychiatric phenotype in women with TXS. Nielsen et al. suggested [43] that future studies should use brain tissue to explore the pathogenetic mechanisms behind TXS, but brain tissue is unavailable from living humans. We suggest using brains from animals like nonhuman primates or infertile cattle with an extra X chromosome [10] or neuronal tissue generated using human induced pluripotent stem cells, preferably from women with TXS and differences in the psychiatric phenotype. The use of human induced pluripotent stem cells harbors the promise of discovering new treatment options for neuropsychiatric disorders [47].

In summary, women with TXS are vulnerable to developing psychiatric disorders, and women with TXS and impaired social functioning are even more vulnerable. Psychotic disorders, major depression, anxiety disorders, suicidality, and low self-esteem, should be considered in the clinical examination of women with TXS. Clinicians who work with women with impaired social functioning and psychiatric disorders should consider referral to a clinical geneticist. Future research should use a longitudinal design, larger groups of participants and preferably an ecological design. We know that the participants of this study gave their time and efforts to find medical and psychological treatments. Future studies also should develop and evaluate treatments for psychiatric disorders in women with TXS.

## Data Availability

The data that support the findings of this study are available from the corresponding author (M.O.), upon reasonable request.
